# Is It Time to Phase Out the Austin Moore Hemiarthroplasty? A Propensity Score Matched Case Control Comparison versus Cemented Hemiarthroplasty

**DOI:** 10.1155/2016/7627216

**Published:** 2016-03-06

**Authors:** Christian Fang, Rui-Ping Liu, Tak-Wing Lau, Anderson Leung, Tak-Man Wong, Terence Pun, Frankie Leung

**Affiliations:** ^1^Department of Orthopaedics and Traumatology, Queen Mary Hospital, The University of Hong Kong Pokfulam, Hong Kong; ^2^Department of Orthopaedics, No. 2 People's Hospital of Changzhou, Affiliated Hospital of Nanjing Medical University, 29 Xinglong Alley, Changzhou, Jiangsu 213003, China

## Abstract

We compared the Austin Moore hemiarthroplasty versus cemented hemiarthroplasties using a propensity score matched cased control study. For a consecutive cohort of 450 patients with displaced intracapsular neck of femur fractures, 128 matched cases in each group were selected based on age, gender, walking status, nursing home residency, delays in surgery, ASA score, and the Charlson comorbidity score. At a mean follow-up of 16.3 months, we evaluated their outcomes. Significantly more patients with AMA experienced thigh pain (RR = 3.5, 95% CI: 1.67–7.33,  *p* = 0.000), overall complications (RR = 4.47, 95% CI: 1.77–11.3, *p* = 0.000), and implant loosening (RR = 8.42, 95% CI: 2.63–26.95, *p* = 0.000). There were no definite cement related deaths in this series. There was no significant difference in mortality, walking status, and the number of revisions between the groups. We support the routine use of cemented hemiarthroplasty instead of the Austin Moore for treating elderlies with displaced intracapsular neck of femur fractures.

## 1. Introduction

Intracapsular neck of femur fracture is common in the elderly and typically managed by hemiarthroplasty when displaced. The Austin Moore hemiarthroplasty (AMA) was introduced in the 1950s [1]. It is a monobloc cementless hemiarthroplasty system with a nonporous coated collared perforated stem inserted by press fit. Although modern modular porous coated cementless systems and cemented systems have continued to evolve, the AMA remains to be in regular use by developed countries [2].

Despite early reports which suggested that uncemented monobloc stems were prone to mechanical loosening and thigh pain [3–5], they remain to be commonly used. Monobloc stems accounted for nearly one-sixth of the 60000 partial hip replacements for fractures in Australia from 2009 to 2014 [2]. In our region, the AMA remained to be the most used prostheses until 2010 for three main reasons. Firstly, small canal diameters in the local population were common [6], precluding routine usage of the other economical cemented monobloc stems such as the Thompson prosthesis. Secondly, there was insufficient evidence to justify routine use of the more costly modern modular implants [7]. Thirdly, surgeons were concerned about cement related embolic complications and mortalities [8, 9] in frail elderlies.

More recently an additional high quality randomized study by Parker et al. [10] favourably compared cemented monobloc stems against the AMA. It was shown that cemented implants resulted in better function, less pain, and less implant related complications with no apparent increase in the risk of cement related complications. The objective of our study was to reconfirm this by a propensity score (PS) matched case control study in the local population after a resultant change in our healthcare policy.

## 2. Methodology

We retrospectively reviewed a consecutive cohort of patients in a university affiliated tertiary hospital with intracapsular hip fractures admitted from December 2010 to June 2014 treated by primary hemiarthroplasty. All patients with the Enhanced ICD-9-CM diagnosis code of 820 (Fracture of Neck of Femur) and procedure code of 81.52 (partial hip replacement) were queried and retrieved from the hospital operative record database.

Patients with pathological fractures were excluded. 450 patients with hemiarthroplasties were obtained. The cemented group consisted of modular or monobloc or cemented implants, and the AMA group consisted solely of uncemented prosthesis. As mandated by the local hip fracture pathway [11], all procedures were carried out using the posterolateral approach as described by Moore [1]. All patients were allowed immediate weight bearing after surgery and followed a standard in-patient rehabilitation programme. In the first half of our study period, the AMA was routinely used in most patients while cemented modular stems were used routinely in the second half due to a change in healthcare policy.

PS matching [12] was carried out as an effort to negate confounding at patient selection which may have occurred in very active or very frail patients. Except for the date of surgery, possible confounding variables in affecting decision making were matched between the two groups. These factors were age, sex, preinjury walking state, residency in a nursing home, Charlson comorbidity score [13] (CCS), American Society of Anaesthesiology (ASA) score, and days of delay in surgery. SPSS (Version 23, IBM, Armonk, USA), R (Version 3.10, The R Foundation, Vienna, Austria), and the Thoemmes algorithm [14] (version 3.04) were used for propensity matching and statistical analysis. The standard nearest-neighbour, one-to-one technique was used with a caliper value of 0.2 [15]. Between the two groups, all confounding variables were compared before and after PS matching to ensure that a standardized mean difference of 0.25 or less was obtained and also that none of these baseline variables were significantly different statistically (Table 1).

The territory wide electronic healthcare database which covered all public hospitals and 95% of the local population was reviewed for each patient. The outcome measures were compared under three categories. They were mortality, clinical outcome, and complications. Mortality data was recovered from the territory wide death registry for all patients who were analysed. Only patients with clear clinical documentation of their functional status after rehabilitation were analysed for clinical outcomes. The three research assistants responsible for charting of clinical outcomes were blinded from the type of prosthesis. Four authors assessed the radiological outcomes and complications and each result was cross verified between two authors. Only patients with radiological follow-up of more than 90 days or those with an earlier known complication were included for radiological and complication analysis.

The radiological complications were implant loosening, defined as a continuous radiolucent line of more than 2 mm which surrounded the stem, stem subsidence of more than 5 mm, or gross varus or valgus displacement. Other complications included deep or superficial wound infections, dislocations, intraoperative cracks, and postoperative traumatic and atraumatic periprosthetic fractures. All revision surgeries were recorded.

The Pearson Chi square test was used for dichotomous variables with frequent occurrences and the Fisher exact test was used for variables with small occurrences. The two-tailed *t*-test was used for continuous variables. A *p* value of less than 0.05 was taken as statistically significant. Comparisons between the two groups before and after PS score matching were listed in the results of the study. The relative risk (RR) with 95% confidence intervals and the number needed to treat (NNT) was calculated for significant outcomes with AMA being the group exposed to risk.

## 3. Results

From the database query, there were 146 cemented implants including 114 collarless polished tapered modular cemented stems with a bipolar or monopolar head and 32 cemented monobloc collared hemiarthroplasties. There were 304 uncemented AMAs in the other group. After propensity score matching, there were 128 patients with a cemented stem and 128 matched patients with an AMA. The mean interval for latest clinical follow-up was 20.7 months and the mean interval for latest X-ray was 16.5 months.

In the matched cemented and AMA group, respectively, the mean age was 80.7 and 81.0. There were no differences between the two groups in terms of gender, premorbid walking ability, residence in a nursing home, average delay from admission to surgery, CCS, and ASA scores. For above factors the maximum standardized mean difference was 0.093, indicating two very closely matched groups.

**Figure 1:**
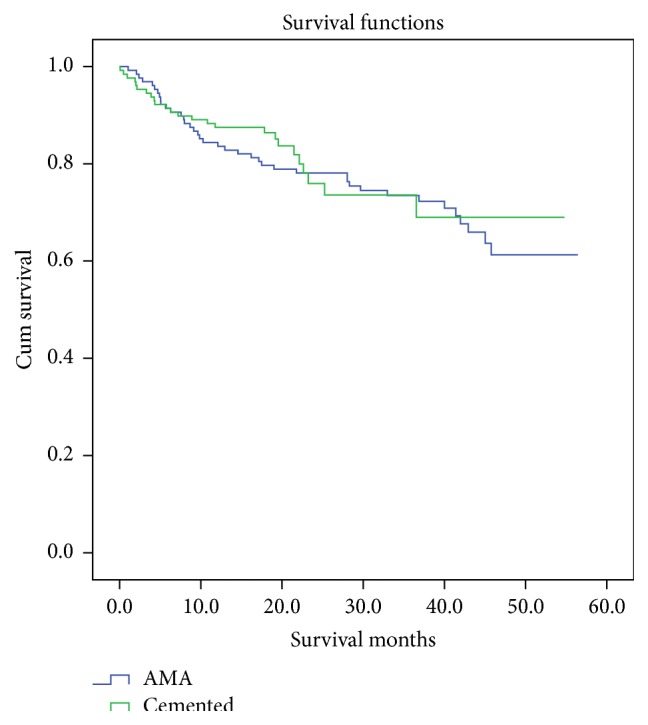
Patient survival until latest follow-up. Log rank test *p* = 0.621.

The mean duration of surgery was 67.7 minutes for a cemented hemiarthroplasty and 56.1 minutes for an AMA (Pearson Chi Square significance, *p* = 0.000). There was no statistical difference in the percentage of patients receiving general anaesthesia or receiving blood transfusions. There was no statistically significant difference in cumulative mortality at 1, 3, 6, and 12 months, as well as till the latest follow-up. The mean estimated survival was 43.1 months for both AMA and cemented implants (resp., 95% CI: 39.6–46.6 and 39.0–47.3, log rank test *p* = 0.621).  Figure 1 shows patient survival until latest follow up.

One out of three patients with cemented arthroplasty who died within one month had a cause of death related to respiratory failure. This patient had chronic obstructive airway disease and died two days after surgery without an autopsy. We observed no deaths within one month after surgery in the matched group of patients who received the AMA.

Including those with an earlier known mortality or known complication, 79.7% (102) patients with cemented hemiarthroplasty and 82% (105) patients were included into analysis of complications. In these patients, 4.2% (5) cemented and 18.9% (23) AMAs, respectively, had a surgical complication (double sided Fisher's exact test, *p* = 0.000). 2.5% (3) cemented and 19.7% (24) AMAs (double sided Fisher's exact test, *p* = 0.000) had definite radiological evidence of implant loosening or subsidence of more than 5 mm before one year. One and four patients, respectively, had a posterior dislocation of the prosthesis. One and three patients, respectively, had deep infection. The differences in incidence of dislocation and infection were not statistically significant (double sided Fisher's exact test, *p* = 0.369 and *p* = 0.621, resp.).

One cemented hemiarthroplasty and seven AMAs had an intraoperative crack (double sided Fisher's exact test, *p* = 0.056). Additionally, there were five cracks in the cemented group as a result of an intended AMA insertion which led to conversion to a cemented system without further consequence, and these were not counted as a complication in either group. Cerclage wires were placed for all seven cracks which occurred at AMA insertion and five of them still had eventual implant loosening.

3.4% (4) of cemented hemiarthroplasties versus 6.6% (8) of AMAs received one or more reoperations (double sided Fisher's exact test, *p* = 0.238). In the cemented group, this included one open reduction and internal fixation of periprosthetic fracture, one conversion to a long stemmed total hip arthroplasty for aseptic loosening, one debridement procedure of deep infection, and one open reduction of an incarcerated posterior dislocation with sciatic nerve palsy. For the AMA group, there were three stem revisions for aseptic loosening, two reentries for cerclage wiring of missed intraoperative cracks, one multistaged revision for septic loosening, one debridement procedure of deep infection, and one Girdlestone procedure for aseptic loosening with dislocation.

7.1% (8) of cemented hemiarthroplasties patients versus 24.8% (29) of AMAs experienced clinically significant thigh pain or hip pain at any time point during the follow-up period; this difference was statistically significant (double sided Fisher's exact test, *p* = 0.000).

88.3% (113) of cemented hemiarthroplasty and 91.4% (117) of AMAs had adequate clinical data to grade their maximum walking status after rehabilitation. In terms of walking status, there was no statistically significant difference between the two groups.

In summary of the significant findings, compared to patients with a cemented hemiarthroplasty, patients with an AMA were 3.5 times more likely to have thigh pain (RR: 95% CI: 1.67–7.33), NNT = 5.6 (95% CI: 3.7–11.8), 4.47 (RR: 95% CI: 1.77–11.30) times more likely to have complications, NNT = 5.9 (95% CI: 3.8–12.5), including 8.42 (RR: 95% CI: 2.63–26.95) times more likely to have implant loosening up to latest follow-up, NNT = 4.6 (95% CI: 3.2–7.8), and 7.77 times (RR: 95% CI: 2.41–25.01), NNT = 5.0 (95% CI: 3.5–9.0), more likely to have implant loosening within one year after surgery. Moreover, patients with an AMA had an increased but statistically insignificant trend in having more revisions, intraoperative cracks, dislocations, and infections.

In our locality, the cost difference between a cemented hemiarthroplasty and an AMA is around USD$400. Based on the calculated NNT, cemented hemiarthroplasty would potentially be cost saving if the following expenditures are exceeded in managing each bad outcome: thigh pain (NNT = 5.6, USD$2240), any complications (NNT = 5.9, USD$2360), and loosening (NNT = 4.6, USD$1840). However, since cost analysis was not the main objective of the current study, the above calculations can only be taken as approximate estimations.

## 4. Discussion

In this study we were able to sufficiently prove that routine usage of cemented stems resulted in improved outcome of patients within one year after surgery. Our findings agreed well with the two previous randomized studies [5, 10].

After a number of unmatched studies which suggested inferiority of the AMA against cemented hemiarthroplasties [3, 4, 8, 16], Sonne-Holm et al. [5] were the first to publish a Randomized Clinical Trial (RCT) of 112 patients comparing the uncemented AMA against the cemented AMA in 1982. It was concluded that cementation of the same prostheses resulted in significantly better pain relief and gait function. Despite that, the practice of cementing an AMA was not widely accepted because of its design intent and worries of extreme difficulty in removal due to cement interlocking with a perforated stem. Twenty years later, Parker et al. performed another high quality RCT with 400 patients comparing the cemented Thompson against the uncemented AMA. The cemented group had less pain and better mobility [10]. These studies have heavily influenced a number of following meta-analyses [17–19], where authors also conclude that cementation improved pain and function. Our study is the third such comparative study that used matched data of AMAs versus cemented hemiarthroplasties.

One patient who received a cemented prosthesis in our study died from cardiopulmonary related complications two days after surgery. However, since an autopsy was not carried out, it was uncertain whether this may have been related to cement thromboembolic events. The risk of cementation remains to be important in patients prone to cardiopulmonary and cerebrovascular compromise [9]. Our study and the other RCTs are likely underpowered to reiterate the small but significant (0.3% versus 0.04%, Pearson Chi square, *p* = 0.02) risk of intraoperative deaths observed from 8639 cemented and 2477 uncemented hemiarthroplasties in the Norwegian hip registry [8].

As shown by other studies, the cemented prosthesis usually resulted in better walking function in addition to thigh pain. This finding however was only reflected in our results (Table 2) before PS matching and mitigated after matching. We therefore believe that walking outcome is more affected by the patient's premorbid status. Patients with poor cognitive status and limited rehabilitation potential may have similar walking function regardless of the implant being used.

More patients had intraoperative cracks during implantation of AMAs. Despite only having borderline statistical significance in this study, it is noteworthy that five additional cracks that were not counted as complications in the cemented group actually resulted from intended AMA implantations. This is in agreement with other studies [8, 17, 20], showing that cementless hemiarthroplasties are associated with more iatrogenic fractures in osteoporotic patients.

It is uncertain here whether better leg length, offset, and soft tissue tension restoration provided by modular cemented implants may have resulted in a small but insignificant trend of less dislocations in cemented implants. It is also unknown from our results whether routine use of gentamicin loaded antibiotic cement may have led to a small but insignificant trend of less infections in cemented hips.

It should be noted that older cementless monobloc stems such as the AMA are distinct from modern cementless modular porous coated prosthesis. The monobloc stems have a number of notable shortcomings. Firstly, the AMA's nonmodular design does not allow for adjustment of the femoral neck length and total hip conversions must be performed with a stem exchange. Secondly, the AMA has a matt and nonporous coated surface finish, providing less surface friction and less early stability and no opportunity for bone ingrowth when compared to porous coated stems. Thirdly, the AMA has a concave medial and convex lateral stem profile and comes in only two available sizes, providing less reliable fitting and three-point stability when compared to double or triple tapered modern stems that are made to fit a variety of canals. These fundamental design differences should be considered when interpreting the conclusions from a number of recent meta-analyses [17–19] which generally compared cemented and cemented hemiarthroplasties and were heavily influenced by the inferior outcomes of the older cementless monobloc stems.

In a number of more recent studies, modern porous coated cementless hemiarthroplasties had mixed outcomes when compared against cemented implants. Rogmark et al. [21] observed inferior cementless implant survival at five years and more periprosthetic fractures in patients older than 75 years but not younger patients in the Norway and Swedish national registry. Langslet et al. [22] demonstrated in an RCT that while modern porous coated cementless hemiarthroplasties had a higher risk of periprosthetic fractures, patients had better hip scores after 5 years. Two other RCTs by Talsnes et al. [23] and Deangelis et al. [24] demonstrated similar functional outcome and low complication rates for modern modular cemented and cementless designs in the first year. In all, not all of the newer studies agreed with each other and the older studies with cementless monobloc stems. Better powered studies and renewed meta-analysis are needed. It is also possible that subgroups of patients may benefit differently from specific modern cemented or uncemented prosthesis.

There are a number of limitations in this study. Firstly, this is a retrospective nonrandomized study. We attempted to negate confounding by PS matching. As there were many cognitively impaired patients who were unable to give consent for an RCT, this may be the next best way to do a comparison study. PS matching has been used in a large number of clinical studies and is becoming increasingly popular [12, 25]. The caliper nearest-neighbour matching technique is one of the standard methods in obtaining optimal balance in moderate to large samples [15]. The main drawbacks of propensity score matching are the trade-off in statistical power and systemic failure when some important confounding factors are overlooked. In our study we eliminated 194 cases. The findings may not apply to the frailest and most active patients as they may have been excluded during matching. This also reduced this study's statistical power in detecting less remarkable differences.

Secondly, the group of cemented hemiarthroplasties was not homogenous. It comprised patients with cemented Thompsons, cemented AMAs, and cemented collarless polished tapered modular stems with monopolar or bipolar heads. Nonetheless, we viewed them as a single group based on the current literature which suggested minimal to no difference between these cemented implants [26, 27]. Lastly, the clinical outcome analysis in this study was partially flawed because standardized scoring systems were not used and 19% of patients either died or had insufficient clinical documentation at follow-up.

## 5. Conclusion

Cemented hemiarthroplasties outperformed the AMAs in terms of pain control, implant stability, and complication rate. We detected no increased early mortality related to cementing complications. We support the routine use of cemented hemiarthroplasties for geriatric intracapsular hip fractures.

## Figures and Tables

**Table 1:** Group characteristics and balance before and after PS matching.

Balance before and after PS matching													
	Before PS matching							After PS matching					
	Cemented		AMA		SMD	*p* value		Cemented		AMA		SMD	*p* value
	146 cases		304 cases					128 cases		128 cases			
	Means	*n* or (range, SD)	Means	*n* or (range, SD)				Means	*n* or (range, SD)	Means	*n* or (range, SD)		
Age	79.0	(36.9–98.2, 8.7)	84.4	(53.7–101, 7.1)	0.616	0.000		80.7	(62.6–98.2, 7.2)	81.0	(53.7–95.9, 7.1)	0.031	0.759
Age above 80	47.9%	70	74.7%	227	0.619	0.000		54.7%	70	56.3%	72	0.032	0.802
Males	28.8%	42	29.9%	91	0.026	0.799		27.3%	35	31.3%	40	0.086	0.492
Independent walker	52.1%	76	34.5%	105	0.349	0.000		46.9%	60	44.5%	57	0.047	0.707
Nonfunctional walker	4.8%	7	10.9%	33	0.283	0.034		5.5%	7	5.5%	7	0.000	1.000
Nursing home residence	13.0%	19	23.7%	72	0.316	0.008		14.1%	18	17.2%	22	0.093	0.491
Days delay in operation	2.1	(0–72, 6.0)	1.8	(0–44, 3.0)	0.057	0.426		2.1	(0–72, 6.4)	2.2	(0–44, 4.4)	0.008	0.946
3 days delay in operation or more	11.6%	17	9.5%	29	0.071	0.490		10.2%	13	12.5%	16	0.069	0.554
ASA score	2.5	(1–4, 0.6)	2.7	(1–4, 0.5)	0.331	0.001		2.5	(1–4, 0.6)	2.5	(1–4, 0.6)	0.000	1.000
ASA score 3 or above	46.6%	68	66.1%	201	0.411	0.000		49.2%	63	51.6%	66	0.046	0.708
Charlson Comorbidity Score	2.1	(0–13, 2.8)	2.6	(0–14, 2.6)	0.180	0.063		2.1	(0–13, 2.8)	2.0	(0–9, 2.2)	0.031	0.787
Charlson Comorbidity Score 5 or above	14.4%	21	21.1%	64	0.164	0.091		14.1%	18	13.3%	17	0.023	0.856
Length of clinical follow-up	17.0 m	(0–53.2, 11.8)	22.8 m	(0.1–55.3, 14.9)		0.000		16.7 m	(0–53.2, 11.6)	24.7 m	(0.4–55.3, 15.2)		0.000
at least 3 months	85.6%	125	81.3%	247		0.252		85.2%	109	82.0%	105		0.500
at least 9 months	65.8%	96	72.4%	220		0.151		64.8%	83	75.0%	96		0.076
Length of radiological follow-up	14.3 m	(0–53.2, 11.6)	17.2 m	(0–55.3, 14.0)		0.030		14.0 m	(0–53.2, 11.4)	19.0 m	(0–55.3, 15.0)		0.003
at least 3 months	77.4%	113	70.7%	215		0.136		76.6%	98	70.3%	90		0.258
at least 9 months	50.7%	74	56.3%	171		0.267		50.8%	65	59.4%	76		0.167

Pearson Chi square test for dichotomous variables.

Two-tailed Independent sampled *t*-test for continuous variables.

(SMD) Standardized Mean Difference = difference in means ÷ Standard deviation.

**Table 2:** Outcomes compared between cemented versus AMA groups before and after PS matching.

Outcomes before and after PS matching												
	Before PS matching					After PS matching					Relative risk (95% CI)	NNT (95% CI)
	Cemented		AMA		*p* value	Cemented		AMA		*p* value		
	146 cases		304 cases			128 cases		128 cases				
	Mean	*n* or (range, SD)	Mean	*n* or (range, SD)		Mean	*n* or (range, SD)	Mean	*n* or (range, SD)			
Duration of Surgery	69.1 mins	(21–171, 23.6)	53.7 mins	(20–135, 21.4)	0.000	67.7 mins	(21–139, 22.5)	56.1 mins	(21–135, 21.6)	0.000		
General anaesthesia	26.7%	36	17.4%	53	0.022	25.8%	33	18.0%	23	0.131		
Received blood transfusion	35.6%	52	38.2%	116	0.602	35.9%	46	32.0%	41	0.510		
Died at 1 month	2.1%	3	3%	9	0.577	2.3%	3	0.0%	0	0.082		
Died at 3 months	4.8%	7	7.9%	24	0.225	4.7%	6	3.1%	4	0.521		
Died at 6 months	8.2%	12	11.8%	36	0.244	8.6%	11	8.6%	11	1.000		
Died at 12 months	12.3%	18	19.1%	58	0.074	12.5%	16	15.6%	20	0.474		
Total length of stay (mean, SD)	29.3 days	(2–292, 26.9)	29.6 days	(5–130, 16.8)	0.880	29.8 days	(2–292, 28.4)	31.8 days	(12–130, 18.0)	0.508		

Fulfill clinical analysis criteria	88.4%	129	86.5%	263	0.585	88.3%	113	91.4%	117	0.410		

post-op functional walker	83.7%	108	74.9%	197	0.053	81.4%	92	80.3%	94	0.868		
post-op recreational walker	21.7%	28	5.3%	14	0.000	15.9%	18	9.4%	11	0.165		
Unable to maintain walking function	42.6%	55	44.9%	118	0.746	45.1%	51	49.6%	58	0.512		
post-op thigh pain	6.2%	8	22.4%	59	0.000	7.1%	8	24.8%	29	0.000	3.50 (1.67–7.33)	5.6 (3.7–11.8)

Fulfill complication analysis criteria	80.1	117	81.3	247	0.779	79.7%	102	82.0%	105	0.635		

Cracks from attempted AMA	4.3%	5				4.9%	5					
Complications	4.3%	5	21.9%	54	0.000	4.9%	5	21.9%	23	0.000	4.47 (1.77–11.30)	5.9 (3.8–12.5)
Loosening	2.6%	3	23.1%	57	0.000	2.9%	3	24.8%	26	0.000	8.42 (2.63–26.95)	4.6 (3.2–7.8)
Loosening before 1 year	2.6%	3	21.9%	54	0.000	2.9%	3	22.9%	24	0.000	7.77 (2.41–25.01)	5.0 (3.5–9.0)
Revision surgeries	3.4%	4	6.9%	17	0.233	3.9%	4	7.6%	8	0.374		
Intraoperative crack	0.9%	1	5.3%	13	0.043	1.0%	1	6.7%	7	0.065		
Dislocations	0.9%	1	3.2%	8	0.282	1.0%	1	3.8%	4	0.369		
Infections	0.9%	1	2.8%	7	0.445	1.0%	1	2.9%	3	0.621		
Traumatic periprosthetic fractures	0.9%	1	1.2%	3	1.000	1.0%	1	0.0%	0	0.493		

NNT: number needed to treat.

Double sided Fisher's exact test for dichotomous variables.

Two-tailed Independent sampled *t*-test for continuous variables.
